# A Robust and Accurate Non-rigid Medical Image Registration Algorithm Based on Multi-level Deformable Model

**Published:** 2017-12

**Authors:** Yanli WAN, Hongpu HU, Yanli XU, Quan CHEN, Yan WANG, Dongping GAO

**Affiliations:** 1. Institute of Medical Information, Chinese Academy of Medical Sciences, Beijing, China; 2. Medical College of Hebei Engineering University, Handan, Hebei, China

**Keywords:** Non-rigid registration, Medical image, Deformable model

## Abstract

**Background:**

Compared to the rigid image registration task, the non-rigid image registration task faces much more challenges due to its high degree of freedom and inherent requirement of smoothness in the deformation field. The purpose was to propose an efficient coarse-to-fine non-rigid medical image registration algorithm based on a multilevel deformable model.

**Methods:**

In this paper, a robust and efficient coarse-to-fine non-rigid medical image registration algorithm is proposed. It contains three level deformation models, i.e., the global homography model, the local mesh-level homography model, and the local B-spline FFD (Free-Form Deformation) model. The coarse registration is achieved by the first two level models. In the global homography model, a robust algorithm for simultaneous outliers (error matched feature points) removal and model estimation is applied. In the local mesh-level homography model, a new similarity measure is proposed to improve the robustness and accuracy of local mesh based registration. In the fine registration, a local B-spline FFD model with normalized mutual information gradient is employed.

**Results:**

We verified the effectiveness of each stage of the proposed registration algorithm with many non-rigid transformation image pairs, and quantitatively compared our proposed registration algorithm with the HBFFD method which is based on the control points of multi-resolution. The experimental results show that our algorithm is more accurate than the hierarchical local B-spline FFD method.

**Conclusion:**

Our algorithm can achieve high precision registration by coarse-to-fine process based on multi-level deformable model, which ourperforms the state-of-the-art methods.

## Introduction

Medical image registration is one of the most important and challenging research in the modern medical image analysis field. It aims to align two images which are captured from different device/time into the same coordinate system. “It has many potential and important applications in clinical diagnosis, such as fusion of computer tomography (CT) and magnetic resonance imaging (MRI) data to obtain more complete information about the patient, monitoring tumor growth, treatment verification, and comparison of the patient’s data with anatomical atlases” (1).

From the view of the image transformation, medical image registration can be classified into rigid registration and non-rigid registration. In the past few years, a number of excellent rigid image registration methods were proposed and widely applied, such as the cross-correlation method (2), maximization of mutual information method (3) and normalized mutual information method (4). These methods are extended or integrated, and gradually applied to solve the non-rigid image registration problem (5–7). Compared to the rigid image registration task, the non-rigid image registration task faces much more challenges due to its high degree of freedom and inherent requirement of smoothness in the deformation field. The accuracy, robustness and speed of these algorithms are required to be further improved for clinical applications (8–9).

The non-rigid medical image registration algorithm naturally depends on the geometric deformation model and the similarity measure criterion. The geometric deformation models can be classified into two main categories: i) physics-based models such as the elastic body models (10–11), the optical flow models (12) and the diffusion models (13); and ii) interpolation-based models such as free-form deformations (14–15). Christensen et al. (10) proposed an approach to tackle large deformations with multiple linear elastic models which represent a series of small deformations. Guyader et al. (11) proposed an approach that combines segmentation and registration that is based on nonlinear elasticity. Lu et al. (12) proposed a registration method based on the optical flow model for non-rigid heart images. This algorithm includes two steps, i.e., coarse registration and precise registration, to improve both of the registration accuracy and the convergence speed. Thirion (13) regarded the image registration as a diffusion process. This algorithm is actually an iterative process between estimation of the pixel displacements and the update of the transformation. In each iteration, the movement of any pixel is decided by a matching process based on the Sum of Squared Differences (SSD) criterion. For the above registration algorithms based on the physical models, it is difficult to construct a reasonable physical model that can simulate the complex tissue deformations between the two input images. Rueckert et al. (14) proposed a local deformation model for non-rigid registration on breast MR images. This model was described by the so called Free-Form Deformation (FFD) based on B-splines, and it employed the normalized mutual information (NMI) as the similarity function. Since the degree of freedom of the local deformation model is determined by the number of control points, it is important to decide whether a sparse or dense set of control points should be used. However, both sparse and dense sets have limitations. If a sparse set of control points is used, the movements of the control points will not well represent complicated deformations. If a dense set of control points is used, the optimization can be computationally inefficient. In order to tackle these shortcomings, some researchers proposed some compromise methods. For example, Shi et al. (15) proposed a multi-level B-spline model in which only a sparse subset of the control points is active to balance speed and accuracy.

In addition to the geometric deformation model itself, extraction of the robust and precise feature correspondences is also very important. It is an essential step to estimate the geometric deformation model in many registration methods. However, it is often affected by image noise, feature outliers and local deformations. In the past decade, a number of methods were proposed to solve the robust feature matching problem. Among them, one kind of fuzzy correspondence methods, such as softassign methods (16) and relaxation labeling methods (17) have been developed, in which the binary constrains of the correspondences are relaxed to become a fuzzy correspondence during the optimization process. Some researchers combined the iconic feature and the geometric feature for correspondence searching and outlier discarding (18–19). However, most of these approaches have limited capability in handling outliers caused by feature extraction errors or large deformations.

This paper proposes an efficient coarse-to-fine non-rigid medical image registration algorithm based on a multi-level deformable model.

## Materials and Methods

The study was carried out according to the Helsinki Declaration and approved by the ethical committee of Chinese Academy of Medical Sciences. The need for informed consent was waived, because the data sets used in this study downloaded from some open web sites.

### Algorithm Overview

**Fig. 1:**
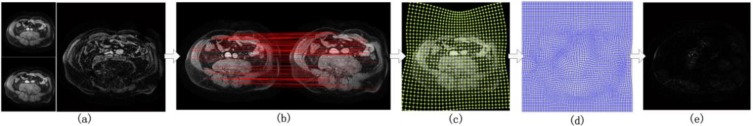
The flow chart of our algorithm

Fig. 1 shows the flow chart of our algorithm, which contains three steps. The left part of Fig. 1 (a) shows the two input images to be registered, where the top one is called the reference image (which will be fixed during the registration step) and the bottom one is called the float image (which will be transformed by the registration process). The right part of (a) shows the difference of the two input images. (e) shows the difference between the fixed reference image and the registered float image after the proposed coarse-to-fine registration algorithm. Comparing (e) and the right part of (a), it can be seen that most pixels of the two input images are already aligned accurately. (b), (c) and (d) are the intermediate results corresponding to the three steps of the proposed coarse-to-fine registration algorithm, and will be described in detail in the following text.

[1] Feature correspondence detection based on global homography model. The robust and accurate feature correspondence detection between the reference image and float image plays an important role in image registration (shown in Fig 1 (b)). In this paper, we used the SIFT (Scale-invariant feature transform) (20–21) algorithm to detect sparse feature correspondences in the two images. Although SIFT can be invariant to uniform scaling, orientation, and partially invariant to affine distortion and illumination changes, it inevitably produces outliers due to feature extraction errors or large deformations. To tackle this issue, an improved robust RANSAC algorithm for simultaneous removal of outliers and estimation of the global level transformation model is applied.
[2] Coarse registration based on local-mesh level homography model Since the global transformation model cannot precisely simulate the local deformations of non-rigid tissue, a number of local deformable models corresponding to a series of uniform grid mesh are robustly estimated. In this step, the local deformation model is estimated by a local homography with shape-preserving constraints. The local deformation mesh is shown in Fig 1 (c). Then, the coarse registration based on the local mesh-level homography model is performed. It can greatly improve the convergence speed and precision of the following fine registration step.
[3] Fine registration based on B-spline FFD model. Although the above local homography transformation model can effectively simulate the deformations to some extent, its ability is limited for the images with very complex deformations because the model reduces degree of freedom by introducing a shape-preserving constraint. To tackle the complex deformations, a fine registration step is further applied. In this step, a B-spline FFD model is constructed by integrating the normalized mutual information (NMI) gradient to acquire more accurate registration results in this step (shown in Fig. 1 (d)).

### Estimation of the Global Level Homography Model

The global level transformation model is described by a homography *H_g_*, a 3 × 3 matrix. Suppose {x_*i*_, x′_*i*_} is the i-th matched SIFT feature correspondence between the reference image R and the float image F, *H_g_* can be estimated by four pairs of correspondences:

[1] $${\text{x}}_{i}^{\prime }=H_{g}{\text{x}}_{i},\;\;i=1,\ldots ,4$$

where x_*i*_ is a homogeneous coordinate, i.e., x_*i*_ =(*x_i_* *y_i_* 1)^*T*^.

**Fig. 2:**
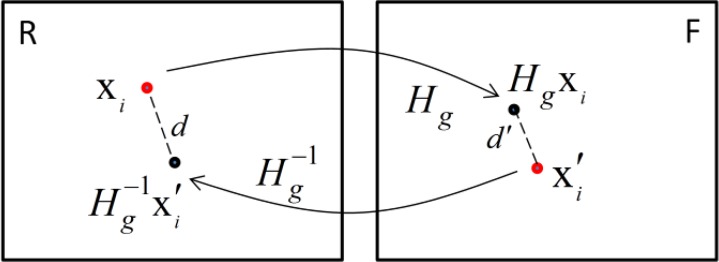
Residual error terms for global homography estimation

In order to enhance the robustness and accuracy of the feature correspondences, the robust ORSA (Optimized Random Sample Consensus, i.e., Optimized RANSAC) algorithm (23) is applied to estimate the parameters of the global homography model *H_g_* and eliminate the outliers simultaneously. The average residual error ε of the correspondences is used to distinguish the inliers and outliers in the process of the estimation (Fig 2).

[2] $$\varepsilon =\frac{1}{2n}\sum_{i=1}^{n}\left(d(x_{i},H_{g}^{-1}{\text{x}}_{i}^{\prime })+d({\text{x}}_{i}^{\prime },H_{g}x_{i})\right)$$

where *d*(x′*_i_*,*H_g_*x_*i*_) is the Euclidean distance between the locations of point x′*_i_* and transformed point *H_g_*x_*i*_.

After performing the ORSA algorithm, a global transformation model *H_g_* and an inlier set (with a relatively large error threshold ε_1_) are obtained. In order to further eliminate outliers, the ORSA algorithm is further applied on the 4 × 4 sub-images of the reference image R with a relatively small error threshold ε_2_. The estimated global homography matrix *H_g_* and the inliers will be used in the following local mesh-level deformation step.

### Coarse Registration based on Local Mesh-level Deformable Model

#### Estimation of the Local Mesh-level Deformable Model

**Fig. 3:**
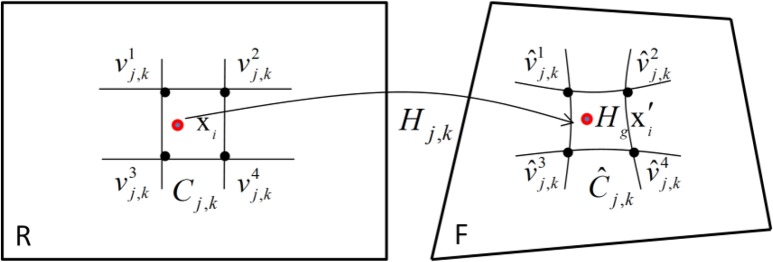
Local deformable model in the corresponding grid cell of the reference image *R* and the float image *F*

The reference image R is divided into a number of regular square cells (called meshes). Each grid cell has β × β (β = 16 in our experiments) pixels. As shown in Fig 3, the grid cell *C_j,k_* is enclosed by 4 vertices $C_{j,k}=[v_{j,k}^{1},v_{j,k}^{2},v_{j,k}^{3},v_{j,k}^{4}]$, and its corresponding cell *Ĉ_j,k_* in the float image F is ${\hat{C}}_{j,k}=[{\hat{v}}_{j,k}^{1},v_{j,k}^{2},v_{j,k}^{3},v_{j,k}^{4}]$. Suppose x_*i*_ is one feature point in cell *C_j,k_*, where *j* = ⌊*x_i_*/β⌋ and *k* = ⌊*y_i_*/β⌋ is the coordinates of the cell. The motion model from *C_j,k_* to *Ĉ_j,k_* can be represented by a local homography *H_j,k_*, i.e., there exists the following linear equation:

[3] $${\hat{C}}_{j,k}=H_{j,k}C_{j,k}$$

Since the local model *H_j,k_* can be linearly solved with the 4 vertices of the grid cell, so the estimation of the local homography model becomes the estimation of the 4 vertices *Ĉ_j,k_*.

Suppose {x_*i*_, x′*_i_*} is the *i*-th feature correspondence between the reference image *R* and float image *F*, then take into account the global homography model *H_g_* that estimated in the first step, we use *H_g_*x′*_i_* to replace x′*_i_* in the estimation of the local homography model, because after global homography transformation, *H_g_*x′*_i_* is more close to x_*i*_ than the original point x′*_i_*. The x_*i*_ and *H_g_*x′*_i_* are separately located in the grid cell *C_j,k_* and *Ĉ_j,k_*. The feature point x_*i*_ can be represented by a 2D bilinear interpolation of the cell *C_j,k_*, i.e., x_*i*_ = C_*j,k*_ω_i_, where ω_*i*_ is the interpolation weight, and there is $\omega_{i}=[\omega_{i}^{1},\omega_{i}^{2},\omega_{i}^{3},\omega_{i}^{4}]$, $\sum_{t=1}^{4}\omega_{i}^{t}=1$. It is supposed that the corresponding point *H_g_*x′*_i_* has the same weight ω_*i*_ in the cell *Ĉ_j,k_* where it is located. Then the data term used to estimate all cell’s vertices *Ĉ* in the float image is defined as:

[4] $$E_{d}(\hat{C})=\sum_{i}\left\|C_{j,k}\omega_{i}-H_{g}{\text{x}}_{i}^{\prime }\right\|$$

Although this flexible motion model can well describe the transformation between the local corresponding cells, it is difficult to estimate it because there are no sufficient feature correspondences in each cell. In order to address this challenge, a similarity constraint term is introduced to limit the degrees of freedom of the motion model:

[5] $$E_{c}(\hat{C})=\sum_{\hat{v}}\left\|\hat{v}-v_{1}-sR_{90}(v_{0}-v_{1)}\right\|,R_{90}=\left[\begin{array}{cc} 0 & 1\\ -1 & 0 \end{array}\right]$$

where *ν̂*, *ν*_0_, *ν*_1_ are three vertices of one grid in the float image, *s* = ‖*ν* − *ν*_1_‖/‖*ν*_0_ − *ν*_1_‖ is a scale ratio computed from the corresponding three vertices in the reference image, and *R*_90_ is a rotation matrix used to guarantee the right angle of vector *ν̂*_1_*ν* and *ν̂*_1_*ν*_0_. This constraint term requires the triangle formed by three neighboring vertices *ν*, *ν*_0_, *ν*_1_ to follow a similarity transformation.

The data term and constraint term are linearly combined to obtain the final energy function:

[6] $$E(\hat{C})=E_{d}(C)+\lambda E_{c}(C)$$

where *λ* is a weight to control the contribution of the two terms. Since the energy function is quadratic, all cell’s vertices *Ĉ* can be solved by a sparse linear solver. Then the local model *H_j,k_* will be estimated by Eq.(3). Note that the final local homography *Ĥ_j,k_* in each cell is determined by *H_j,k_H_g_*.

The data term in the above mentioned energy function mainly considers the position information of the feature correspondences. In order to further improve the robustness of the local homography estimation algorithm, a new similarity measure with stronger constraints by employing direction and distance information (See Sec.4.2) is proposed for adaptively determining the weight *λ* of the constraint term in Eq.(6). The weight *λ* is equally discretized into 10 values between 0.3 and 3. Taking the center point of each grid as the center of the support window in the reference image, the corresponding support window in the float image can be determined by the estimated local homographies. Then the similarity is calculated between the corresponding windows, and the optimal local homographies are selected as those with maximum similarity measure.

Once the optimal local homographies *Ĥ_j,k_* are determined, they are used for coarse registration by transforming and interpolating the float image to obtain the coarsely registered image *F′*. It is worth noting that after the transformation, the pixel coordinates are mapped to the non-integer coordinates, so the pixel interpolation is the key step in the registration. In this paper, we use the partial volume interpolation method (PVI) (24). PVI is one kind of interpolation method that is based on the joint histogram, and can obtain a more smooth objective function curve.

#### A New Similarity Measure based on NCC

The new similarity measure is a weighted normalized cross correlation (Weighted NCC) aiming to select the optimal local homographies. As we know, NCC is a simple but effective similarity measure. However, it only uses the gray pixel values to measure the similarity of the corresponding local windows. In fact, direction of the pixel gradient is also valuable and powerful information for similarity measurement. In this paper, a new similarity measure is proposed by assigning a weight for each pixel except the center point in a support window. It is defined as:

[7] $$C(\text{x},{\text{x}}^{\prime })=\frac{\sum_{i}w(\text{x}+i)w^{\prime }({\text{x}}^{\prime }+i)(I(\text{x}+i)-\bar{I}(\text{x}))(I^{\prime }({\text{x}}^{\prime }+i)-{\bar{I}}^{\prime }({\text{x}}^{\prime }))}{\sqrt{\sum_{i}w^{2}(\text{x}+i{)(I(\text{x}+i)-\bar{I}(\text{x}))}^{2}\sum_{i}{w^{\prime }}^{2}({\text{x}}^{\prime }+i){\left(I^{\prime }({\text{x}}^{\prime }+i)-{\bar{I}}^{\prime }({\text{x}}^{\prime })\right)}^{2}}}$$

where *I*(x) and *I′*(x′) are the gray values at point x and x′ in the image *R* and *F* respectively. *Ī*(x) and *Ī′*(x′) are the mean gray pixel values in the given β × β windows centered at x and x′. In Eq.(7), the weights *w*(x+*i*) and *w′*(x′+*i*) are respectively determined by the product of two components of direction and distance, i.e. *w_r_*(x+*i*) * *w_s_*(x+*i*) and *w′_r_*(x+*i*) * *w′_s_*(x+*i*). The following section takes weight *w*(x+*i*) as an example to introduce the meaning of the two components.

The direction component is computed as:

[8] $$w_{r}(\text{x}+i)=\text{exp}(-\left\|\psi (\text{x}+i)-\psi (\text{x})\right\|/\delta_{r})$$

where ψ(x+*i*) and ψ(x) are the main direction at point x + *i* and x. ‖ψ (x + *i*)−ψ (x)‖ is the angle between the two main directions. δ*_r_* is a scale factor. The main direction, such as ψ(x), is determined by the histogram of oriented gradient (HOG) formed by the gradient orientations of all pixels within a 8×8 window region centered at x(*x*, *y*). The orientation histogram is equally discretized into 36 bins covering 360 degree range of orientations. The gradient orientations of each pixel in the corresponding bin of histogram θ(*x*, *y*) are added by its gradient magnitude *m*(*x*,*y*):

[9] $$\{\begin{array}{l} m(x,y)=\sqrt{{\left(L(x+1,y,\sigma )-L(x-1,y,\sigma )\right)}^{2}+{\left(L(x,y+1,\sigma )-L(x,y-1,\sigma )\right)}^{2}}\\ \theta (x,y)={\text{tan}}^{-1}\left(\left(L(x,y+1,\sigma )-L(x,y-1,\sigma )\right)/\left(L(x+1,y,\sigma )-L(x-1,y,\sigma )\right)\right)\\ L(x,y,\sigma )=G(x,y,\sigma )*I(x,y) \end{array}$$

where the Gaussian window *G*(*x*,*y*,σ) (σ = 2.5 in our experiments) aims to give emphasis on gradients close to the center of the region. The peak value of the orientation histogram is considered as the main direction. Then the angle of two main directions can be calculated by the number of bins of histogram.

Similarly, the distance component is computed as:

[10] $$w_{s}(x+i)=\text{exp}(-d(x+i,x)/\delta_{s})$$

where *d*(**x** + *i*, **x**) is the Euclidean distance between the locations of pixel x + *i* and x. δ_*s*_ is a scale factor and it is set as the half of the support window, i.e., β/2. The pixel with smaller distance to the center of the region will be assigned a higher weight in Eq.(10).

In a word, the new similarity measure presents stronger constraints by combing direction and distance information to weight each pixel in the support window. It can obtain the best score when each pixel has similar direction and distance simultaneously.

#### Fine Registration based on B-spline FFD Model

In order to better adapt to the local complex deformation of organ tissue, an additional transformation is required. We chose an FFD model, based on B-splines, which affects the transformation only in the local neighborhood of the control point. Suppose image *F′* is divided into a number of mesh cells and let ϕ denote a *n_s_* × *n_y_* mesh of control points φ_*i,j*_ with uniform spacing δ (δ = 4 in our experiments). The FFD can be defined as:

[11] $$T(x,y)=\sum_{l=0}^{3}\sum_{m=0}^{3}B_{l}(u)B_{m}(v)\varphi_{i+l,j+m}$$

where (*x*, *y*) is a point in the image *F′*. *i* = ⌊*x*/δ⌋ − 1, *j* = ⌊*y*/δ⌋ − 1, *u* = *x*/δ − ⌊*x*/δ⌋, *ν* = *y*/δ − ⌊*y*/δ ⌋, and *B_i_* represents the *l*th basis function of the B-spline:

[12] $$\{\begin{array}{l} B_{0}(u)={\left(1-u\right)}^{3}/6\\ B_{1}(u)=\left(3u^{3}-6u^{2}+4\right)/6\\ B_{2}(u)=(-3u^{3}+3u^{2}+3u+1)/6\\ B_{3}(u)=u^{3}/6 \end{array}$$

To find the optimal local transformation, a cost function which consists of smoothing constraint term *C*_*B*_*FFD*_ and similarity measure term *C*_*NMI*_ is defined as follows:

[13] $$C(\phi )=-C_{NMI}(N_{1},T(N_{2}))+\mu C_{B\_FFD}(T)$$

where μ (μ = 0.01 in our experiments) is a weight to balance the two terms of the cost function. *C*_*B*_*FFD*_ (*T*) is used to guarantee the smoothness of the spline-based FFD transformation, and *C*_*NMI*_(*N*_1_,*T*(*N*_2_)) measures the similarity by the normalized mutual information. Because (*x*, *y*) is only affected by its 4 × 4 neighboring control points, i.e. the position of the control point φ_*i,j*_ is only depends its 4δ × 4δ neighborhood grid. So NMI is not calculated on the full image, and it is only calculated between the neighborhood of the corresponding control point (i.e.,*N*_*1*_ and *T*(*N*_2_)) before and after transformation. This will greatly improve the computational efficiency. The two terms of the energy function defined in Eq.(13) are given as follows:

[14] $$\{\begin{array}{l} C_{B\_FFD}(T)=\frac{1}{S}\int_{0}^{w}\int_{0}^{h}\left[{\left(\frac{\partial^{2}T}{\partial x^{2}}\right)}^{2}+2{\left(\frac{\partial^{2}T}{\partial x\partial y}\right)}^{2}+{\left(\frac{\partial^{2}T}{\partial y^{2}}\right)}^{2}\right]dxdy\\ C_{NMI}\left(N_{1},T(N_{2})\right)=\frac{H(N_{1})+H(T(N_{2}))}{H(N_{1},T(N_{2}))} \end{array}$$

where *S* is the area of the image domain, and *w* and *h* are its width and height. *H*(*N*_1_) and *H*(*T*(*N*_2_)) denote the marginal entropy at *N*_1_ and *T*(*N*_2_), and *H*(*N*_1_,*T*(*N*_2_)) denotes their joint entropy.

The key of the fine registration algorithm is to find the optimal transformation parameters ϕ by minimizing the cost *C*(ϕ). We employ an iterative gradient descent optimization method which steps in the direction of the gradient vector with a certain step size ρ. The procedure of the algorithm is summarized in Algorithm 1.

Algorithm 1. The fine registration algorithm.

Step 1: Partition the coarse registration image *F′* to initialize the control point set ϕ.
Step 2: Calculate the gradient of the cost function in Eq.(13), i.e. $\nabla C=\frac{\partial C}{\partial \phi }$.
Step 3: If ‖∇*C*‖≥ξ (ξ = 10^−4^ in our experiments), update the control point set $\phi =\phi +\rho \frac{\nabla C}{\left\|\nabla C\right\|}$, and turn to step 2.
Step 4: Compute *T*(*x*, *y*) by Eq.(11), Obtain the fine registration image *F*″ by the interpolation method (PVI).

## Results

### Algorithm Validation

**Fig. 4:**
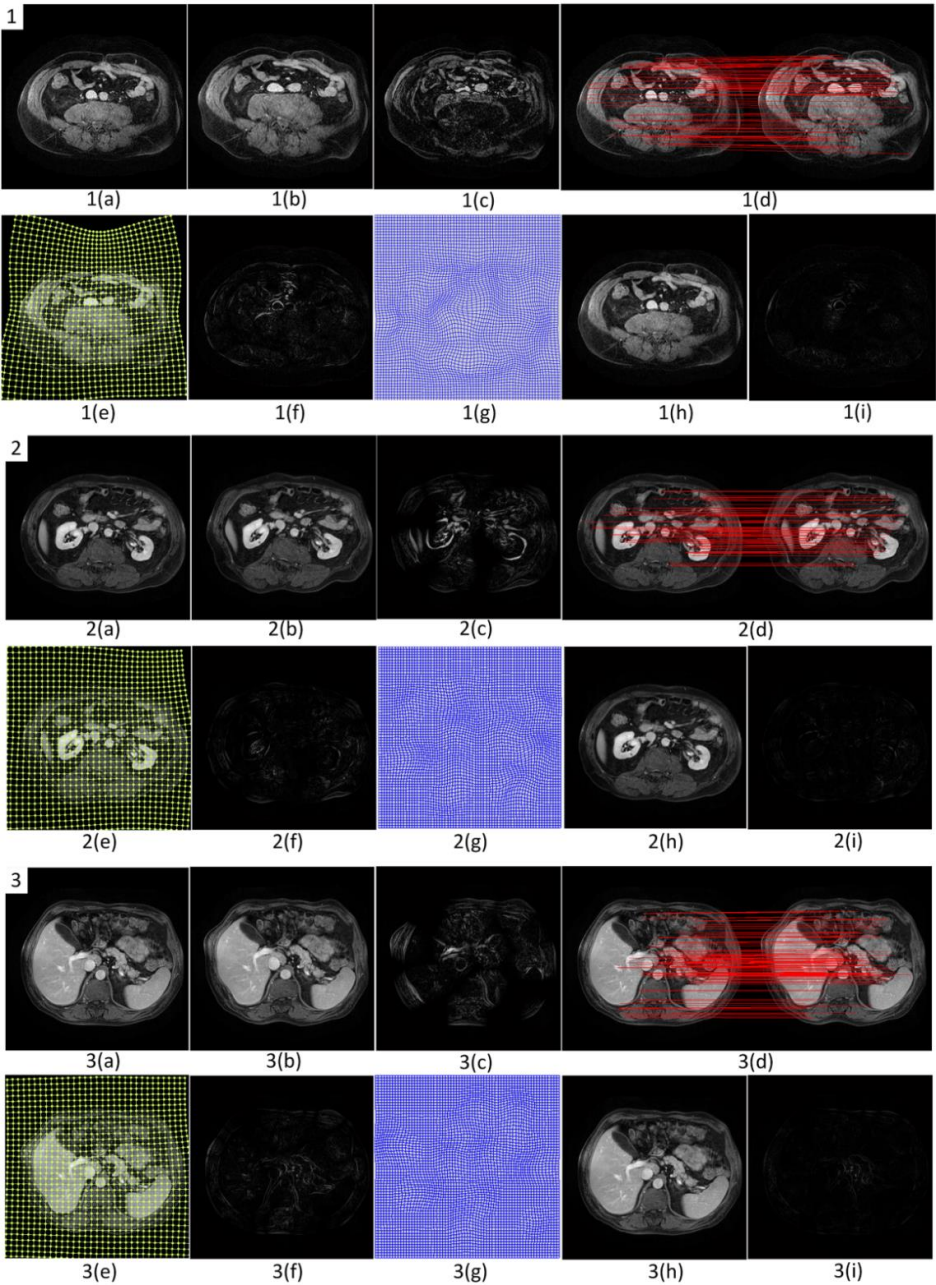
Three sets of experimental results produced by our coarse-to-fine registration algorithm

We first verify the effectiveness of each stage of the proposed registration algorithm with many non-rigid transformation image pairs. Fig. 4 shows the results of three brain data sets after performing the registration algorithm, where (a) is the reference image (image download from (25), ARRA project) and (b) is the float image. The difference of (a) and (b) is shown in (c). From the difference we can see that there are large local deformations between the two input images. (d) shows the correspondences after outliers discarding by robustly estimating the global homography transformation model. (e) shows the estimated warpped meshes from all inliers. The warped meshes are represented by a series of local homographies which are used to perform the coarse registration. The difference between the float image and the coarsely registered image is shown in (f). It demonstrates that the deformation becomes smaller after performing the coarse registration. (g) is the transformation mesh which is determined based on B-spline FFD model. (h) is the deformed image after the fine registration. (i) shows the difference between the reference image (a) and the final registered float image (h). A large number of experiments show that, although there are the large local deformations between the two images, a very small difference can be obtained by performing our coarse-to-fine non-rigid medical image registration algorithm which means accurate registration can be achieved.

At the same time, our method can achieve fast convergence in the fine registration stage because a coarse registration step is applied first to compensate the large displacements.

**Fig. 5:**
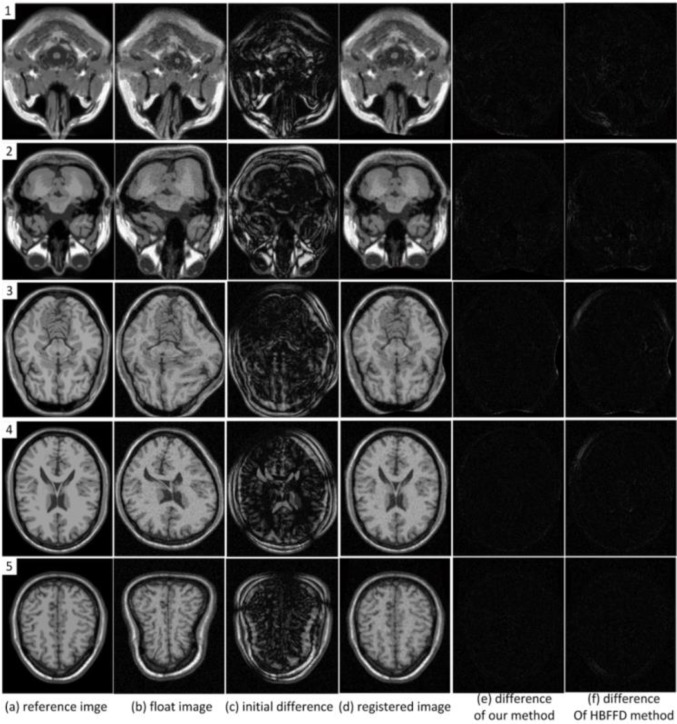
Five sets of experimental results produced by our coarse-to-fine registration algorithm

Fig (5) shows another set of testing results to demonstrate the effectiveness of the proposed algorithm. This dataset has strong noises and large deformations (26). In Fig. (5), (a) and (b) are the reference images and float images, (c) shows the difference of images in (a) and (b). (d) and (e) show the registered float images and the differences with the reference images. (f) shows the difference of the reference and the float images registered by the hierarchical local B-spline FFD method (called HBFFD method). Obviously, our algorithm has better robustness to noises and transformations than HBFFD method.

### Quantitative Comparison and Evaluation

We also quantitatively evaluate and measure the proposed registration algorithm and compare it with the HBFFD method which is based on the control points of multi-resolution. The similarity measurements used to evaluate the accuracy of our algorithm are sum of squared differences (SSD), sum of absolute difference (SAD), normalized correlation coefficient (NCC), and normalized mutual information (NMI). Table 1 lists the experimental results of the three data sets shown Fig. 4 and five data sets shown Fig. 5. From the results we can see that our method can obtain better similarity scores between the registered float images and the reference images than HBFFD method on each data set.

**Table 1:** Comparison of HBFFD and our algorithm with different similarity measurements

***Data sets***	***Registration method***	***Similarity***			
		**SSD**	**SAD**	**CC**	**NMI**
Fig (4)-1	HBFFD	0.000139	0.007298	0.997311	1.362331
	Our method	0.000094	0.004915	0.999132	1.427246
Fig. (4)-2	HBFFD	0.000068	0.005895	0.999017	1.440683
	Our method	0.000041	0.003386	0.999611	1.501243
Fig. (4)-3	HBFFD	0.000174	0.007753	0.999326	1.496891
	Our method	0.000132	0.005291	0.999753	1.564322
Fig. (5)-1	HBFFD	0.001145	0.025060	0.990610	1.240709
	Our method	0.001002	0.020108	0.993157	1.252207
Fig. (5)-2	HBFFD	0.003002	0.028780	0.985250	1.242566
	Our method	0.001091	0.018340	0.991436	1.254210
Fig. (5)-3	HBFFD	0.001557	0.027649	0.986869	1.223756
	Our method	0.001241	0.025660	0.989843	1.233242
Fig. (5)-4	HBFFD	0.001321	0.027423	0.991089	1.240364
	Our method	0.000927	0.021150	0.995822	1.251860
Fig. (5)-5	HBFFD	0.001051	0.023837	0.991260	1.242578
	Our method	0.000828	0.020012	0.996651	1.253413

## Discussion

In addition to the geometric deformation model itself, extraction of the robust and precise feature correspondences is also very important. It is an essential step to estimate the geometric deformation model in many registration methods. However, it is often affected by image noise, feature outliers and local deformations. In the past decade, a number of methods were proposed to solve the robust feature matching problem. Among them, one kind of fuzzy correspondence methods, such as softassign methods (16) and relaxation labeling methods (17) have been developed, in which the binary constrains of the correspondences are relaxed to become a fuzzy correspondence during the optimization process. Some researchers combined the iconic feature and the geometric feature for correspondence searching and outlier discarding (18–19). However, most of these approaches have limited capability in handling outliers caused by feature extraction errors or large deformations.

This paper proposes an efficient coarse-to-fine non-rigid medical image registration algorithm based on a multi-level deformable model. Compared to the existing non-rigid medical image registration methods, our algorithm has the following characteristics:

1. The multi-level deformable model consists of global homography model, local mesh-level homography model and local B-spline based FFD model. The coarse registration process which is based on the first two level models can effectively improve the convergence speed. It also helps improve the precision of the fine registration process which employs an iterative optimization model. More reliable registration results can be obtained compared to the hierarchical local B-spline FFD method which is based on the control points of multi-resolution.
2. In order to improve the robustness of the registration algorithm, on the one hand, a robust algorithm for simultaneous outliers removal and model estimation is applied in the estimation of the global level homography model; on the other hand, a new similarity measure with strong constraints is proposed and applied in the local mesh-level homography model. It combines direction and distance information to weight each pixel in a support window, so as to achieve more accurate comparison of corresponding pixels.

## Conclusion

This paper proposes an efficient coarse-to-fine non-rigid medical image registration algorithm based on a multi-level deformable model. The multi-level deformable model consists of global homography model, local mesh-level homography model and local B-spline based FFD model. In the estimation of the global level transformation model, a robust algorithm for simultaneous outliers removal and model estimation is applied. A new similarity measure with strong constraints is proposed to robustly estimate the local mesh-level deformable model. It combines the direction and distance information to weight each pixel in the support window. The coarse registration of the first two level models can greatly improve the convergence speed and help improve the precision of the fine registration stage. The experimental results show that our algorithm is more accurate than the hierarchical local B-spline FFD method which is based on the control points of multi-resolution.

## Ethical considerations

Ethical issues (Including plagiarism, informed consent, misconduct, data fabrication and/or falsification, double publication and/or submission, redundancy, etc.) have been completely observed by the authors.
